# Comparative cost assessment of the Kato-Katz and FLOTAC techniques for soil-transmitted helminth diagnosis in epidemiological surveys

**DOI:** 10.1186/1756-3305-3-71

**Published:** 2010-08-14

**Authors:** Benjamin Speich, Stefanie Knopp, Khalfan A Mohammed, I Simba Khamis, Laura Rinaldi, Giuseppe Cringoli, David Rollinson, Jürg Utzinger

**Affiliations:** 1 Department of Epidemiology and Public Health, Swiss Tropical and Public Health Institute, Basel, Switzerland; 2 University of Basel, Basel, Switzerland; 3 Helminth Control Laboratory Unguja, Ministry of Health and Social Welfare, Zanzibar, United Republic of Tanzania; 4 Department of Pathology and Animal Health, Faculty of Veterinary Medicine, University of Naples 'Federico II', Regional Center for Monitoring Parasites (CREMOPAR) Regione Campania, Naples, Italy; 5 Wolfson Wellcome Biomedical Laboratories, Department of Zoology, Natural History Museum, London, UK

## Abstract

**Background:**

The Kato-Katz technique is widely used for the diagnosis of soil-transmitted helminthiasis in epidemiological surveys and is believed to be an inexpensive method. The FLOTAC technique shows a higher sensitivity for the diagnosis of light-intensity soil-transmitted helminth infections but is reported to be more complex and expensive. We assessed the costs related to the collection, processing and microscopic examination of stool samples using the Kato-Katz and FLOTAC techniques in an epidemiological survey carried out in Zanzibar, Tanzania.

**Methods:**

We measured the time for the collection of a single stool specimen in the field, transfer to a laboratory, preparation and microscopic examination using standard protocols for the Kato-Katz and FLOTAC techniques. Salaries of health workers, life expectancy and asset costs of materials, and infrastructure costs were determined. The average cost for a single or duplicate Kato-Katz thick smears and the FLOTAC dual or double technique were calculated.

**Results:**

The average time needed to collect a stool specimen and perform a single or duplicate Kato-Katz thick smears or the FLOTAC dual or double technique was 20 min and 34 sec (20:34 min), 27:21 min, 28:14 min and 36:44 min, respectively. The total costs for a single and duplicate Kato-Katz thick smears were US$ 1.73 and US$ 2.06, respectively, and for the FLOTAC double and dual technique US$ 2.35 and US$ 2.83, respectively. Salaries impacted most on the total costs of either method.

**Conclusions:**

The time and cost for soil-transmitted helminth diagnosis using either the Kato-Katz or FLOTAC method in epidemiological surveys are considerable. Our results can help to guide healthcare decision makers and scientists in budget planning and funding for epidemiological surveys, anthelminthic drug efficacy trials and monitoring of control interventions.

## Background

Chronic infections with one or several of the common soil-transmitted helminths, *Ascaris lumbricoides*, *Trichuris trichiura *and the hookworms (*Ancylostoma duodenale *and *Necator americanus*), might account for a global burden of 39 million disability-adjusted life years (DALYs) lost annually [1,2]. School-aged children in the developing world are at highest risk of morbidity due to soil-transmitted helminthiasis.

In the current era of 'preventive chemotherapy', that is the large-scale administration of anthelminthic drugs to school-aged children and other populations at risk of morbidity [3], diagnosis is often neglected and cost-effectiveness considerations are necessary. Yet, diagnosis is of paramount importance for an accurate assessment of the epidemiological situation and burden of disease estimations, and for monitoring drug efficacy and pharmacovigilance [4-7]. In epidemiological surveys pertaining to soil-transmitted helminthiasis (and intestinal schistosomiasis), the Kato-Katz technique [8] is a widely used diagnostic approach [2,9-11]. Indeed, the method is relatively straightforward, requires minimal equipment which is mostly reusable, and hence the method is thought to be inexpensive [7,10]. Moreover, the Kato-Katz method is simple to apply and laboratory workers can be trained within half a day [12]. A drawback of the Kato-Katz method, however, is its lack of sensitivity for detecting light-intensity soil-transmitted helminth infections [9,13]. New research has revealed that the recently developed FLOTAC technique [14] shows a higher sensitivity than multiple Kato-Katz thick smears for the diagnosis of soil-transmitted helminth infections [15-17]. Compared to the Kato-Katz method, the FLOTAC is a more complicated technique, and hence requires better equipped laboratories [18-20] and more extensive training of laboratory workers.

Here, an economic evaluation of the Kato-Katz and FLOTAC techniques was performed within the frame of an epidemiological survey. Specifically, the costs related to stool collection, transfer to the laboratory, preparation and microscopic examination of single and duplicate Kato-Katz thick smears and the FLOTAC dual or double technique were determined. This information is relevant for researchers and disease control managers for the planning of epidemiological surveys and the monitoring and evaluation of soil-transmitted helminthiasis control programmes.

## Materials and methods

### Study area, population and ethical considerations

This study was carried out on Unguja, the largest island belonging to the Zanzibar archipelago in Tanzania, between March and May 2009. The two primary schools of Kinyasini and Kilombero, located 26 and 32 km north-east of Zanzibar Town, were the selected field sites. Laboratory examinations were conducted in the Helminth Control Laboratory Unguja (HCLU), in Zanzibar Town. The study was readily embedded in a randomised controlled trial, assessing the efficacy and safety of four anthelminthic drug regimens against *T. trichiura *and other soil-transmitted helminths. At the beginning of the study, 1,066 schoolchildren aged between 6 and 20 years, were screened for soil-transmitted helminth infections (Knopp S, Mohammed KA, Speich B, Hattendorf J, Khamis IS, Khamis AN, Stothard JR, Rollinson D, Marti H, Utzinger J: "Albendazole and mebendazole administered alone or in combination with ivermectin against *Trichuris trichiura*: a randomized controlled trial", submitted).

The study was approved by the ethics committee of Basel, Switzerland (EKBB; reference no. 13/09) and the Ministry of Health and Social Welfare (MoHSW) of Zanzibar (reference no. ZAMEC/0001/09). Parents or legal guardians of participating children signed a written informed consent sheet. Children consented orally to participate. At the end of the study, all children attending the primary schools of Kinyasini and Kilombero were treated with single oral doses of albendazole (400 mg) and praziquantel (40 mg/kg) free of charge.

### Field and laboratory procedures

The headmasters of Kinyasini and Kilombero primary schools were informed about the purpose and procedures of the study. After having obtained written informed consent by the parents/legal guardians and oral consent by the children, the field work was launched. Every morning, starting at 07:00 hours at HCLU, stool containers and collection shelves were loaded onto a 4-wheel drive (4-WD) car, and a team consisting of 4-6 workers from HCLU visited either Kinyasini or Kilombero schools. The team labeled empty containers with unique identification (ID) numbers, distributed these to the children and collected filled containers that had been distributed the day before. Each day, approximately 100 children were enrolled and lime-sized early morning stool samples were collected.

The filled containers were transferred to HCLU within 2-3 h and processed as follows. Immediately after arrival at HCLU, duplicate Kato-Katz thick smears from each stool sample were prepared by 2-8 members of HCLU, using 41.7 mg templates [8]. After a clearing time of 20-40 min, each Kato-Katz thick smear was examined quantitatively for hookworm eggs by one of four experienced microscopists. In the afternoon, 3-6 h after slide preparation, the thick smears were re-examined by one of four additional microscopists, who counted eggs of *A. lumbricoides *and *T. trichiura *and recorded them separately.

Once a Kato-Katz thick smear had been prepared, the stool sample was placed back into the collection shelf ordered by increasing ID. A third of the stool samples was taken and a small amount of stool was weighed to ~1 g using a Kern balance (EMB basic balance; Ballingen-Frommern, Germany) and preserved in a pre-labeled tube containing 10 ml of 5% formaldehyde. The preserved stool samples were stored at room temperature and examined by the FLOTAC technique within 3 weeks after completion of the clinical trial in late May 2009 [14].

While technicians from HCLU were already acquainted with the Kato-Katz technique, a 3-day training course was offered for the FLOTAC technique, facilitated by two experts from the University of Naples, Italy. During the training, the FLOTAC dual technique was employed, using flotation solutions 4 (FS4; sodium nitrate, NaNO_3_, specific gravity (s.g.) = 1.20) and 7 (FS7; zinc sulfate, ZnSO_4_·7H_2_O, s.g. = 1.35).

The individual steps for FLOTAC preparation and microscopic examination of stool samples were performed by 8-12 members of HCLU and are detailed in Table 1. Preliminary results showed that FS4 outperformed FS7 for the diagnosis of soil-transmitted helminths, and hence FS4 was used throughout. Individual stool samples were prepared according to the FLOTAC dual technique [14]. In brief, ~1 g of stool was equally distributed to the two chambers of the FLOTAC apparatus, but instead of using two different flotation solutions, FS4 was applied in both chambers. Hence, the reading of the chambers was performed according to the FLOTAC basic technique [14].

**Table 1:** Working steps and number of time measurements to determine the costs of the Kato-Katz and FLOTAC method for the diagnosis of soil-transmitted helminths in Zanzibar.

Working step	n	**Number of measurements**^a^
**Kato-Katz (K-K) measurements**		
**(K1a) **Labeling of K-K slides	109	19 × 5; 2 × 4; 1 × 6
**(K1b) **Preparation of duplicate K-K slides	137	121 × 1; 6 × 0.5; 5 × 2; 1 × 3
**(K2) **Microscopy morning	341	32 × 1; 22 × 2; 22 × 8; 21 × 4; 1 × 5
**(K3) **Microscopy afternoon	396	44 × 1; 26 × 8; 24 × 2; 24 × 4
**(K6a) **Washing of K-K slides	180	1 × 180
**(K6b) **Washing spatula and template	297	1 × 91; 1 × 96; 1 × 110
**(K6c) **Cutting cellophane paper	82	1 × 82
**(K6d) **Cutting wire mesh	206	1 × 206
**FLOTAC measurements**		
**(F1) **Measurement of 1 g of stool and homogenization	168	29 × 5; 1 × 6; 1 × 7; 1 × 10
**(F2a) **Filter (inclusive labeling)	31	31 × 1
**(F2b) **Filter (exclusive labeling)	41	25 × 1; 4 × 3; 2 × 2
**(F2c) **Labeling Falcon tubes	33	3 × 6; 2 × 4; 1 × 7
**(F3) **Centrifugation of tubes and filling with FS	62	20 × 3; 1 × 2
**(F4) **Assembling FLOTAC apparatus	29	29 × 1
**(F5) **Filling FLOTAC apparatus	55	55 × 1
**(F6) **Centrifugation and translation of FLOTAC apparatus	36	18 × 2
**(F7) **Reading first chamber	120	120 × 1
**(F8) **Reading second chamber	119	119 × 1
**(F11a) **Washing filter and beaker	82	9 × 1; 9 × 6; 1 × 4; 1 × 7; 1 × 8
**(F11b) **Opening FLOTAC apparatus	52	33 × 1; 1 × 2; 1 × 3; 1 × 14
**(F11c) **Washing FLOTAC apparatus	26	2 × 3; 1 × 2; 1 × 4; 1 × 14
**(F11d) **Washing Falcon tube	208	3 × 4; 2 × 5; 1 × 101; 1 × 84; 1 × 1
**(F11e) **Washing pipettes	200	9 × 1; 1 × 105; 1 × 84; 1 × 1
**Measurements for both techniques**		
**(S1-S3) **Collection of stool samples at school	2,921	^b^
**(K4/F9) **Data entry (demographic data)	13	1 × 13
**(K5/F10) **Data entry (results)	407	5 × 22; 5 × 23; 3 × 20; 3 × 9; 2 × 14; 1 × 5; 1 × 15; 1 × 21; 1 × 26
**(K6f/F11f) **Putting a piece of paper into an empty container to alleviate cleaning of container	138	7 × 10; 1 × 68
**(K6g/F11g) **Removing stool out of container (before cleaning)	260	4 × 10; 2 × 36; 1 × 38; 1 × 52; 1 × 58
**(K6h/F11h) **Washing of stool container (without lid)	65	5 × 10; 1 × 15
**(K6i/F11i) **Washing lid	56	3 × 10; 1 × 5; 1 × 6; 1 × 15
**(K6j/F11j) **Erasing of IDs from stool container	290	18 × 10; 1 × 110

^a ^Interpreted as follows: for example, in the working step "Labeling of K-K slides" it was measured 19 times how much time was required to label 5 slides, 2 times the time for labeling 4 slides and once the time for labeling 6 slides.

^b ^2,921 stool samples were collected in 38 visits at schools, where always a different number of stool samples was collected.

### Cost estimations

**Figure 1:**
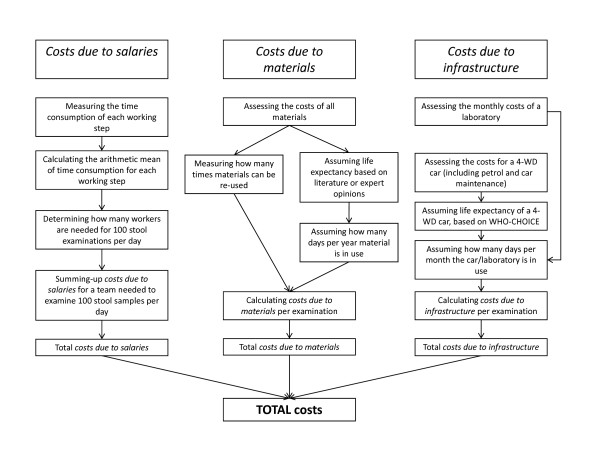
**Flowchart visualizing the determination of the total costs for one stool examination in an epidemiological survey on soil-transmitted helminth infections**.

To assess the costs for single and duplicate Kato-Katz thick smears and the FLOTAC dual or double technique, the following expenses were determined: (i) *costs due to salaries*; (ii) *costs due to materials*; and (iii) *costs due to infrastructure*. We made the following assumptions: screening of 3,000 children for soil-transmitted helminth infections by an experienced research team, with 100 stool samples examined per day in a laboratory (Appendix). The flow of cost determination is shown in Figure 1. All costs are reported in 2009 US$.

#### *Costs due to salaries*

To assess the *costs due to salaries*, the salary of each member of the HCLU, according to his or her professional degree, was determined using a questionnaire. Additionally, the time for each working step in the field and in the laboratory was measured several times and averaged. Finally, the number of employees with a specific occupation needed to collect, process and examine 100 stool samples per day using the Kato-Katz or FLOTAC technique was calculated.

For the collection of stool samples in the field the following steps were recorded: (i) the time from departure in the laboratory, school visit and return to the laboratory; (ii) the number of personnel who visited the school; and (iii) the number of stool samples collected per day. Using these data, the average person-time to collect one stool sample was estimated.

**Figure 2:**
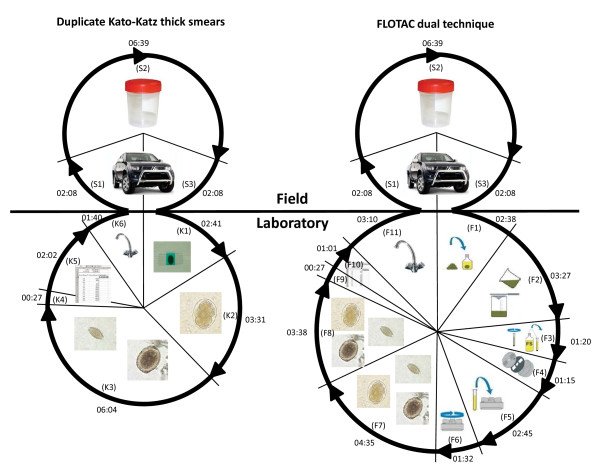
**Time (min:sec) to perform duplicate Kato-Katz thick smears or the FLOTAC dual technique in an epidemiological survey on soil-transmitted helminth infections**. The area of each segment of the circles is proportional to the amount of time used in each working step. S1: Driving to school. S2: Distribution of pre-labeled stool container, and collection of stool samples. S3: Driving to laboratory. K1: Labeling microscope slides and preparing duplicate Kato-Katz thick smears. K2: Quantitative microscopic reading for hookworm eggs K3: Quantitative microscopic reading for *A. lumbricoides *and *T. trichiura *eggs. K4/F9: Data entry (name, age, sex and school grade). K5/F10: Data entry (egg counts from Kato-Katz/FLOTAC). K6/F11: Washing and preparing materials for the next day. F1: Weighting ~1 g of stool and homogenization in 10 ml 5% formaldehyde. F2: Filtering homogenized stool and filling into two Falcon tubes. F3: Centrifugation of the Falcon tubes, discarding supernatant and filling flotation solutions into tubes. F4: Assembling of the FLOTAC apparatus. F5: Filling FLOTAC apparatus with homogenized suspension (pellet and flotation solution). F6: Centrifugation and afterwards translation of FLOTAC apparatus. F7: Reading chamber 1 quantitatively for soil-transmitted helminth eggs. F8: Reading chamber 2 quantitatively for soil-transmitted helminth eggs.

The duration of each distinct step in the laboratory needed to perform the Kato-Katz or FLOTAC technique was measured and averaged (Table 1 and Figure 2). For duplicate Kato-Katz thick smears the steps were as follows: (K1) labeling duplicate microscope slides with specific ID and preparation of duplicate Kato-Katz thick smears; (K2) quantitative microscopic reading of duplicate Kato-Katz thick smears for hookworm eggs; (K3) quantitative microscopic reading of duplicate Kato-Katz thick smears for *A. lumbricoides *and *T. trichiura *eggs; (K4) data entry (children's name, age, sex and school grade); (K5) data entry (results from all Kato-Katz thick smear readings); and (K6) washing and preparing materials for the next day.

For the FLOTAC technique the following steps were included: (F1) weighing ~1 g of stool and homogenization in 10 ml of 5% formaldehyde; (F2) filtering homogenized stool and transfer into two 15-ml Falcon tubes labeled with personal ID; (F3) centrifugation of Falcon tubes, discarding supernatant and filling FS4 into tubes; (F4) assembly of FLOTAC apparatus; (F5) filling each chamber of the FLOTAC apparatus with the homogenized stool suspension (pellet and FS4) from one of the two Falcon tubes; (F6) centrifugation and translation of FLOTAC apparatus; (F7) reading chamber 1 of FLOTAC apparatus quantitatively for *A. lumbricoides*, hookworm and *T. trichiura *eggs; (F8) reading chamber 2 of FLOTAC apparatus quantitatively for *A. lumbricoides*, hookworm and *T. trichiura *eggs; (F9) data entry (children's name, age, sex and school grade); (F10) data entry (results from all FLOTAC readings); and (F11) washing and preparing materials for the next day.

The time to prepare and read a single Kato-Katz thick smear was assessed as follows: (K1a) the time needed to label duplicate microscope slides was divided by 2 (Table 1); (K1b) the time to prepare a single Kato-Katz thick smear was directly measured; (K2), (K3) and (K5) the average time of the working steps was divided by 2; (K6d-j) the time was equal for single or duplicate Kato-Katz thick smears; (K11a-c) the time was divided by 2. The time to prepare and read the FLOTAC double technique was assessed as follows: (F2c), (F3-F6), (F10) and (F11b-e) the average time was divided by 2; (F8) this working step was excluded. In the remaining working steps the measured time was applicable for the FLOTAC dual or double technique.

To calculate the total time expenditure for a single and duplicate Kato-Katz thick smears and the FLOTAC dual and double technique, the arithmetic means of the time needed to perform a specific working step were summed-up. Out of the total working time, it was determined how many employees were needed to perform 100 stool examinations per day, assuming that laboratory staff work 7 h per day. Subsequently, the total salary costs for the team were calculated. The costs were divided by 100, to obtain the *costs due to salaries *per stool examination.

#### *Costs due to materials*

To estimate the total *costs due to materials*, it was differentiated between three kinds of materials: (i) materials that can only be used once (e.g. wooden spatula, wire mesh); (ii) materials that can be reused (e.g. microscope slides and pipettes); and (iii) materials that have a long life expectancy (e.g. microscope and centrifuge). The frequency of possible re-use was determined in the field and laboratory. Additionally, the effort to render the materials re-usable was accounted for as a working step (e.g. washing). The life duration of materials with a longer life expectancy was estimated by consulting laboratory experts and the literature. For these materials, it was estimated how many days per year they were to be used. All materials, asset costs, life time, days in use and total costs are listed in Table 2.

**Table 2:** Price of material needed for duplicate Kato-Katz thick smears and the FLOTAC dual technique, taking into account the asset cost, life time and days of use.

Material	Asset cost (US$)	Present material (n)	Lost material (n)	Examinations per material (n)	Estimated life expectancy (years)	Estimated days in use per year	Price per stool examination (US$)
**FLOTAC dual technique**							
Wooden spatula	0.02						0.02
Falcon tubes (for stool preservation)	0.04						0.04
5% formaldehyde (1 l)	1.25						0.01
0.9% NaCl solution (1 l)	0.20						< 0.01
FS4 (NaNO3, 1 l)	12.11						0.13
Pasteur-pipettes	0.05	716	95	7			0.01
Falcon tubes (for preparation)	0.04			10			0.01
Filter for FLOTAC	9.73	700	0	1,000			0.01
FLOTAC apparatus	29.51			1,000			0.03
FLOTAC apparatus (reading disk)	2.21			100			0.02
Balance (Kern EMB basic balance)	172.57				10	50	< 0.01
Centrifuge (Hettich Universal 320)	5,865.44				10	50	0.12
Centrifuge (Hettich EBA 3 [second hand])	147.56				2	50	0.01
**Duplicate Kato-Katz thick smears**							
Cellophane paper	0.01						0.01
Wire mesh (3 cm^2^)	< 0.01						< 0.01
Microscope slide	0.09	6,000	300	20			< 0.01
Kato-Katz kit (template and plastic spatula)	0.30	974	20	48.7			0.01
**FLOTAC dual technique and duplicate Kato-Katz thick smears**							
Gloves	0.01						< 0.01
Container (120 ml) for stool collection	0.15	3,170	249	12.73			0.01
Hand tally counter	19.43				10	100	< 0.01
Microscope (Olympus CX 21)	938.83				10	100	0.01

#### *Costs due to infrastructure*

*Costs due to infrastructure *originated from (i) the use of the laboratory building for stool examinations (e.g. rent, tap water and electricity); and (ii) the use of the car for stool container distribution and collection in schools. The monthly costs for the laboratory and the asset costs for the 4-WD car, together with the monthly expenditures for car maintenance were reported by the head of HCLU. The life expectancy of a car was derived by consulting WHO-choice [21] and the petrol costs were assessed on the spot in Zanzibar (average diesel price for March-May 2009).

### One-way sensitivity analyses

A series of one-way sensitivity analyses were performed to determine the robustness of the cost estimations and to assess to what extent the costs vary if a specific parameter changes [22-24]. We alternated single parameters pertaining to the total costs of the Kato-Katz or FLOTAC technique. For the scenario indicated in the Appendix, the following parameters were considered: (i) salary; (ii) unproductive time; (iii) *costs due to materials*; (iv) petrol costs; (v) *costs due to infrastructure*; and (vi) costs without collection of stool samples in the field.

## Results

### Parasitological findings and diagnostic sensitivity

Among 1,066 school children screened with duplicate Kato-Katz thick smears, the prevalence of *T. trichiura*, hookworm and *A. lumbricoides *was 62.8%, 19.8% and 9.2%, respectively. The FLOTAC double technique was performed on 343 stool samples and revealed respective prevalences of 67.1%, 11.7% and 10.2%. Infection intensities, according to WHO guidelines [25], were mostly light. Considering the combined results of stool samples examined with duplicate Kato-Katz thick smears and the FLOTAC double technique as diagnostic 'gold' standard, the sensitivity of duplicate Kato-Katz thick smears for detection of *T. trichiura*, hookworm and *A. lumbricoides *eggs was 88%, 81% and 68%, respectively. The respective sensitivities for FLOTAC were 95%, 54% and 88%.

### Cost Outcomes

#### *Costs due to salaries*

In 2009 the daily salary of an employee at HCLU was, on average, US$ 18.83 (minimum: US$ 17.65, maximum US$ 22.18; excluding managers' salaries).

The average person-time needed to collect one stool sample in the field, including transfer to HCLU was 10 min and 55 sec (10:55 min) (Figure 2 [S1-S3]). The average time to prepare and read duplicate Kato-Katz thick smears was 16:26 min (Figure 2 [K1-K6]) and 09:39 min for a single Kato-Katz thick smear, whereas for the FLOTAC dual technique a total of 25:49 min (Figure 2 [F1-F11]) and for the FLOTAC double technique a total of 17:19 min was recorded. The total time for one stool examination, including stool collection in the schools and transfer to HCLU, was 20:34 min for a single Kato-Katz thick smear, 27:21 min for duplicate Kato-Katz thick smears, 28:14 min for the FLOTAC double technique and 36:44 min for the FLOTAC dual technique.

Including the salary of the workers, the *costs due to salaries *for one stool examination were US$ 1.25 for a single Kato-Katz thick smear, US$ 1.57 for duplicate Kato-Katz thick smears, US$ 1.64 for the FLOTAC double technique and US$ 1.95 for the FLOTAC dual technique.

#### *Costs due to materials*

The prices of all materials used for stool examinations at HCLU together with examinations and assumptions about their life expectancy, are summarized in Table 2. The *costs due to materials *for single or duplicate Kato-Katz thick smears were US$ 0.03 and US$ 0.04, respectively. For the FLOTAC double and dual technique the *costs due to materials *were US$ 0.26 and US$ 0.43, respectively.

#### *Costs due to infrastructure*

The monthly costs for the use of the laboratory building were reported to be US$ 256.77. Assuming that 3,000 stool samples were examined during 1.5 months (30 working days), the costs of the laboratory building were US$ 0.13 per stool sample. The 4-WD car was bought in 2002 for US$ 22,000. WHO-choice suggests a lifetime for a 4-WD car of 8 years [21]. Considering that the car is in use at 200 days per year, the costs per day were US$ 31.91 (including monthly car maintenance of US$ 51.87 and daily petrol consumption of 15 l diesel). Assuming that 100 stool samples were collected per day, the costs per sample due to the car were US$ 0.32. Hence, the *costs due to infrastructure *were US$ 0.45 for each diagnostic method in a survey of 30 working days with a total of 3,000 stool samples collected by a research team in a field-setting located ~30 km from the laboratory and examined by a team of experienced laboratory technicians.

#### Total costs

**Figure 3:**
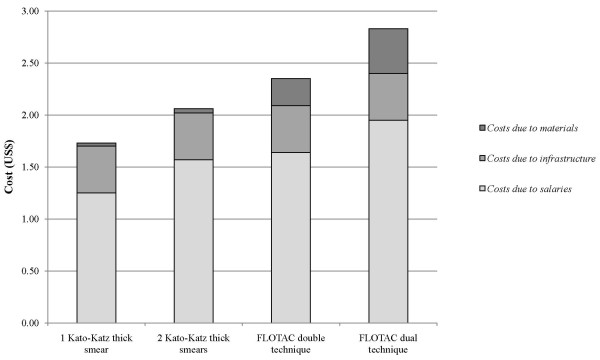
**Total costs for the Kato-Katz and FLOTAC technique for the diagnosis of soil-transmitted helminth infections in an epidemiological survey carried out in Zanzibar in 2009, and taking into account the *costs due to salaries, costs due to materials *and *costs due to infrastructure***.

Taking into account the *costs due to salaries*, *costs due to materials *and *costs due to infrastructure*, the total expenses were US$ 1.73 for a single Kato-Katz thick smear, US$ 2.06 for duplicate Kato-Katz thick smears, US$ 2.35 for the FLOTAC double technique and US$ 2.83 for the FLOTAC dual technique (Figure 3).

### One-way sensitivity analyses

The one-way sensitivity analyses illustrate the effect of alternated cost variables on the total costs of either diagnostic approach (Table 3). If salaries were doubled, the increase in total cost for one stool examination ranged from 68.9% (FLOTAC dual technique) to 76.3% (duplicate Kato-Katz thick smears). If 30% of unproductive time was added to the total time of laboratory working steps, the increase in total costs ranged from 11.9% (single Kato-Katz thick smear) to 18.4% (FLOTAC dual technique). Material costs had a major impact on the total costs of the FLOTAC technique, but not on the total costs of the Kato-Katz method: doubling the *costs due to materials *increased the total costs of the FLOTAC double and dual technique by 11.1% and 15.2%, respectively, while the total costs for the Kato-Katz method increased by less than 2%. Doubling the *costs due to infrastructure *raised the total cost for one stool examination in the range of 15.9% (FLOTAC double technique) to 25.9% (single Kato-Katz thick smear). Doubling the petrol costs raised the total costs by 5.3% (FLOTAC dual technique) to 8.6% (single Kato-Katz thick smear).

**Table 3:** Results of the series of one-way sensitivity analyses to illustrate the impact of alternated cost parameters on the total costs of the Kato-Katz and the FLOTAC method for the diagnosis of soil-transmitted helminth infections.

	Costs in US$ (change in %)			
	
Parameter tested	Single Kato-Katz thick smear	Duplicate Kato-Katz thick smears	FLOTAC double technique	FLOTAC dual technique
**Baseline**	1.73	2.06	2.35	2.83
***Costs due to salaries***				
Salaries increase by 100%	2.99 (+72.3%)	3.64 (+76.3%)	3.99 (+69.8%)	4.78 (+68.9%)
30% of unproductive time per working step in the laboratory	1.94 (+11.9%)	2.41 (+16.8%)	2.72 (+15.8%)	3.35 (+18.4%)
***Costs due to materials***				
Material costs increase by 100%	1.76 (+1.7%)	2.10 (+1.9%)	2.61 (+11.1%)	3.26 (+15.2%)
***Costs due to infrastructure***				
Petrol costs increase by 100%	1.88 (+8.6%)	2.21 (+7.3%)	2.50 (+6.4%)	2.98 (+5.3%)
Infrastructure costs increase by 100%	2.18 (+25.9%)	2.51 (+21.8%)	2.80 (+19.2%)	3.28 (+15.9%)
**Others**				
Stool samples brought to laboratory by participants	0.75 (-56.8%)	1.12 (-45.9%)	1.40 (-40.6%)	1.93 (-31.8%)

In a scenario where stool samples are not collected in the field (costs of car and field-work excluded (Figure 2 [S1-S3])), the total costs decrease in the range of 31.8% (FLOTAC dual technique) to 56.8% (single Kato-Katz thick smear).

## Discussion

The time requirements and costs for the diagnosis of soil-transmitted helminth infections in a cross-sectional epidemiological survey, using either the Kato-Katz technique performed on fresh stool samples or the FLOTAC technique using preserved stool samples, are considerable. Indeed, in our study, the costs for a single or duplicate Kato-Katz thick smears were US$ 1.73 and US$ 2.06, respectively. As expected, the costs for the FLOTAC technique were even higher: US$ 2.35 for the FLOTAC double technique and US$ 2.83 for the FLOTAC dual technique. The higher costs of the FLOTAC technique are mainly caused by the longer preparation time for stool samples and microscopic examinations in the laboratory. Previous studies have shown that the FLOTAC technique is more sensitive than the Kato-Katz method for the diagnosis of soil-transmitted helminth infections [15-18], and hence higher costs might be justified. In the present study, the FLOTAC outperformed the Kato-Katz method only for *A. lumbricoides *and *T. trichiura *diagnosis, but was seemingly less sensitive for hookworm diagnosis, for reasons reported elsewhere (Knopp S, Speich B, Rinaldi L, Mohammed KA, Khamis IS, Mohammed AZ, Albonico M, Rollinson D, Marti H, Cringoli G, Utzinger J: "Diagnostic accuracy of the Kato-Katz and FLOTAC techniques when used for assessing anthelmintic drug efficacy", submitted).

In view of the high costs to collect a single stool sample in the field and subsequent examination in the laboratory, preventive chemotherapy without prior diagnosis, as advocated by WHO for high-risk groups in endemic settings is, at first sight, justified [3,26]. In endemic settings, where morbidity control is shifting to infection and transmission control, an accurate assessment of the epidemiological situation is required and renders diagnosis necessary [5,7]. For evaluating the efficacy of routinely applied and newly developed drugs, as well as for individual patient management, accurate diagnostic tools are undoubtedly needed [5,7]. Whenever diagnosis for soil-transmitted helminthiasis is warranted, our results can be of value to decision makers and scientists in budget-planning for epidemiological surveys and to heads of diagnostic laboratories for patient management.

It is important to note that in our study, the Kato-Katz method was performed by rigorously adhering to the WHO bench aids [27]. Indeed, all Kato-Katz thick smears were read twice, first 20-40 min after preparation for hookworm and 3-6 h later a second time for *A. lumbricoides *and *T. trichiura*. While this procedure increases costs, it results in higher sensitivity of the Kato-Katz method and might explain the differences in the diagnostic performance of the Kato-Katz and FLOTAC techniques compared to previous comparative investigations [15-18]. Routinely, however, Kato-Katz thick smears are read only once for the three common soil-transmitted helminth species, which saves costs, but also decreases sensitivity.

Moreover, our data are derived from a large survey, where many stool samples were collected and processed simultaneously. One-way sensitivity analyses showed that costs decrease by more than 50% if, for example, individual patients are managed at hospital or public health centre laboratories, without a need for a field team and a 4-WD car. However, in those laboratories, stool samples are not necessarily examined in large numbers, and hence cost and time are likely higher than presented here, due to economy of scale issues [20]. Of note, additional expenses for the patient will then arise from travel costs and loss of income due to missed working hours [28].

Furthermore, in our setting, the prevalences of *A. lumbricoides *and hookworm were low and infection intensities of all soil-transmitted helminth species were light. Hence, the counting of helminth eggs by microscopists was relatively quick. In settings with higher prevalence and infection intensities, quantitative microscopic examination will be more time consuming and costs will rise.

Of additional cost relevance is that salaries usually constitute the largest part of the total service costs [29]. Our sensitivity analysis indicates that an alteration of salaries is impacting most on the total costs of both the Kato-Katz and FLOTAC method. In contrast to the Kato-Katz, the total costs of FLOTAC are also strongly influenced by alterations in material costs. It should be noted that the costs for material maintenance (e.g. centrifuge and microscope), the sourcing of chemicals and the adequate disposal of hazardous chemicals were not included in our cost calculations. Moreover, transfer of large equipment (e.g. centrifuge) and costs that might be incurred at customs were not considered in our analyses. Highly setting-specific and dependant on economy are infrastructural costs including rent, electricity and petrol.

In our study, the time to prepare and microscopically examine a stool sample using the FLOTAC double technique was 17:19 min. This result is in line with the time of 21 min reported in a recent study, where the FLOTAC technique was applied for the diagnosis of *Fasciola hepatica *in rats [19]. However, another study applying FLOTAC for the diagnosis of non-human *Trichuris *infections revealed a preparation and reading time of only 09:48 min [20]. Of note, our laboratory workers were newly trained to use the FLOTAC method and it is likely that they might have performed faster if the method was being applied routinely. Hence, costs would have been saved. On the other side, the costs of more extensive training necessary to learn the FLOTAC technique in comparison with the simple training for the Kato-Katz method were not considered in our analyses.

Another potential bias in our study is that that the laboratory workers were constantly supervised by an external researcher and that their working speed was recorded. It is hence possible that the staff's working speed differed from normal activity [29]. It is also widely acknowledged that every employee has unproductive phases during a normal working day [30]. To account for unproductive working time, we added 30% of the total measured working time for all working steps in the laboratory in one of the sensitivity analyses. This increased the total costs of the diagnostic methods by up to 18%. The working steps in the field (Figure 2, S1-S3) were excluded from the calculations since unproductive time was already included in the respective time measurements (time record of departure from, and arrival at, the laboratory). Since it is well known that employees make conscious and unconscious breaks during the working day [30], we consider the cost estimations including 30% of unproductive working time closer to reality. For the reason that in our study workers were observed and because unproductive time was not included in our baseline calculation, our cost estimates are rather conservative.

Despite that the cost estimates for the Kato-Katz and FLOTAC method from our study are not readily transferable to other epidemiological settings where additional costs might occur, or costs might be saved, we believe that they give a reasonable idea of the expenses related to soil-transmitted helminth diagnosis and can guide scientists and decision makers in budget planning for epidemiological surveys. The real costs of diagnosis are of considerable relevance to large-scale control programmes, which often need to balance the costs of treatments against the costs of diagnosis. As global funds to control soil-transmitted helminth infections become more widely available this will be an increasingly important issue to ensure that control programmes are cost-effective and sustainable.

## Competing interests

G. Cringoli is the inventor and current patent holder of the FLOTAC apparatus. In case the currently ongoing validation of the FLOTAC technique for human and veterinary diagnosis will be successful, the method will be licensed free of charge to the WHO and interested public non-commercial research centers. None of the other authors has any conflict of interest concerning the work reported in this paper.

## Authors' contributions

BS, SK, KAM, GC and JU designed the study; BS, SK, KAM, ISK, LR, DR and JU implemented the study; BS managed the data; BS, SK and JU analysed and interpreted the data; BS wrote the first draft of the paper; SK, KAM, ISK, LR, GC, DR and JU revised the manuscript. All authors read, and approved the manuscript prior to submission and assisted with the final revision of the manuscript.

## Appendix

Scenario of epidemiological survey to illustrate the effect of changing cost parameters on the total costs of the Kato-Katz and FLOTAC technique.

### Framework

Screening of stool samples obtained from 3,000 schoolchildren for soil-transmitted helminth infections within 30 working days. One hundred children are screened per day. One working day consists of a maximum of 7 working hours.

### Objective

To assess how much the alteration of a single parameter can influence the total costs of a single or duplicate Kato-Katz thick smears and of the FLOTAC double or dual technique for diagnosing soil-transmitted helminths.

### Method

In a series of one-way sensitivity analyses it was estimated how the total costs for one stool examination with the Kato-Katz or FLOTAC method change under the following assumptions:

• *Costs due to salaries*:

- the salaries of employees are doubled

- 30% of unproductive working time is added to each laboratory working step

• *Costs due to materials*:

- all material costs are doubled

• *Costs due to infrastructure*:

- the petrol price is doubled

- costs of the whole infrastructure is doubled

• Others:

- stool samples are submitted directly to a laboratory (field-related costs from driving to schools and collecting stool samples, as well as the costs for a 4-WD car are omitted)

## References

[B1] Chan MS The global burden of intestinal nematode infections - fifty years on Parasitol Today 1997 13 438 443 10.1016/S0169-4758(97)01144-7 15275146

[B2] WHO Prevention and control of schistosomiasis and soil-transmitted helminthiasis: report of a WHO expert committee WHO Tech Rep Ser 2002 912 1 57 12592987

[B3] WHO Preventive chemotherapy in human helminthiasis: coordinated use of anthelminthic drugs in control interventions: a manual for health professionals and programme managers 2006 World Health Organization, Geneva

[B4] Albonico M Engels D Savioli L Monitoring drug efficacy and early detection of drug resistance in human soil-transmitted nematodes: a pressing public health agenda for helminth control Int J Parasitol 2004 34 1205 1210 10.1016/j.ijpara.2004.08.001 15491582

[B5] Bergquist R Johansen MV Utzinger J Diagnostic dilemmas in helminthology: what tools to use and when? Trends Parasitol 2009 25 151 156 10.1016/j.pt.2009.01.004 19269899

[B6] Hotez PJ Molyneux DH Fenwick A Kumaresan J Ehrlich Sachs S Sachs JD Savioli L Control of neglected tropical diseases N Engl J Med 2007 357 1018 1027 10.1056/NEJMra064142 17804846

[B7] Johansen MV Sithithaworn P Bergquist R Utzinger J Towards improved diagnosis of zoonotic trematode infections in Southeast Asia Adv Parasitol 2010 73 171 195 full_text 20627143 10.1016/S0065-308X(10)73007-4

[B8] Katz N Chaves A Pellegrino J A simple device for quantitative stool thick-smear technique in schistosomiasis mansoni Rev Inst Med Trop Sâo Paulo 1972 14 397 400 4675644

[B9] Booth M Vounatsou P N'Goran EK Tanner M Utzinger J The influence of sampling effort and the performance of the Kato-Katz technique in diagnosing *Schistosoma mansoni *and hookworm co-infections in rural Côte d'Ivoire Parasitology 2003 127 525 531 10.1017/S0031182003004128 14700188

[B10] Kongs A Marks G Verle P Van der Stuyft P The unreliability of the Kato-Katz technique limits its usefulness for evaluating *S. mansoni *infections Trop Med Int Health 2001 6 163 169 10.1046/j.1365-3156.2001.00687.x 11299032

[B11] Enk MJ Lima AC Drummond SC Schall VT Coelho PM The effect of the number of stool samples on the observed prevalence and the infection intensity with *Schistosoma mansoni *among a population in an area of low transmission Acta Trop 2008 108 222 228 10.1016/j.actatropica.2008.09.016 18973744

[B12] WHO Action against worms 2004 4 World Health Organization, Geneva

[B13] Knopp S Mgeni AF Khamis IS Steinmann P Stothard JR Rollinson D Marti H Utzinger J Diagnosis of soil-transmitted helminths in the era of preventive chemotherapy: effect of multiple stool sampling and use of different diagnostic techniques PLoS Negl Trop Dis 2008 2 e331 10.1371/journal.pntd.0000331 18982057 PMC2570799

[B14] Cringoli G Rinaldi L Maurelli MP Utzinger J FLOTAC: new multivalent techniques for qualitative and quantitative copromicroscopic diagnosis of parasites in animals and humans Nat Protoc 2010 5 503 515 10.1038/nprot.2009.235 20203667

[B15] Utzinger J Rinaldi L Lohourignon LK Rohner F Zimmermann MB Tschannen AB N'Goran EK Cringoli G FLOTAC: a new sensitive technique for the diagnosis of hookworm infections in humans Trans R Soc Trop Med Hyg 2008 102 84 90 10.1016/j.trstmh.2007.09.009 18028969

[B16] Knopp S Rinaldi L Khamis IS Stothard JR Rollinson D Maurelli MP Steinmann P Marti H Cringoli G Utzinger J A single FLOTAC is more sensitive than triplicate Kato-Katz for the diagnosis of low-intensity soil-transmitted helminth infections Trans R Soc Trop Med Hyg 2009 103 347 354 10.1016/j.trstmh.2008.11.013 19168197

[B17] Glinz D Kigbafori SD Knopp S Yao P Lohourignon LK Steinmann P Rinaldi L Cringoli G N'Goran EK Utzinger J Comparing diagnostic accuracy of Kato-Katz, Koga agar plate, ether-concentration, and FLOTAC for *Schistosoma mansoni *and soil-transmitted helminths PLoS Negl Trop Dis 2010 4 e754 10.1371/journal.pntd.0000754 20651931 PMC2907416

[B18] Knopp S Glinz D Rinaldi L Mohammed KA N'Goran EK Stothard JR Marti H Cringoli G Rollinson D Utzinger J FLOTAC: a promising technique for detecting helminth eggs in human faeces Trans R Soc Trop Med Hyg 2009 103 1190 1194 10.1016/j.trstmh.2009.05.012 19573886

[B19] Duthaler U Rinaldi L Maurelli MP Vargas M Utzinger J Cringoli G Keiser J *Fasciola hepatica*: comparison of the sedimentation and FLOTAC techniques for the detection and quantification of faecal egg counts in rats Exp Parasitol 2010 126 161 166 10.1016/j.exppara.2010.04.020 20434442

[B20] Levecke B De Wilde N Vandenhoute E Vercruysse J Field validity and feasibility of four techniques for the detection of *Trichuris *in simians: a model for monitoring drug efficacy in public health? PLoS Negl Trop Dis 2009 3 e366 10.1371/journal.pntd.0000366 19172171 PMC2621347

[B21] WHO-choice Choosing interventions that are cost effective (WHO-CHOICE) http://www.who.int/choice/en/accessed: 25 May 2010

[B22] Shin DW Yun YH Choi IJ Koh E Park SM Cost-effectiveness of eradication of *Helicobacter pylori *in gastric cancer survivors after endoscopic resection of early gastric cancer Helicobacter 2009 14 536 544 10.1111/j.1523-5378.2009.00721.x 19889071

[B23] Hiligsmann M Reginster JY Potential cost-effectiveness of denosumab for the treatment of postmenopausal osteoporotic women Bone 2010 47 34 40 10.1016/j.bone.2010.03.009 20303422

[B24] Gordon LG Obermair A Potential hospital cost-savings attributed to improvements in outcomes for colorectal cancer surgery following self-audit BMC Surg 2010 10 4 10.1186/1471-2482-10-4 20105290 PMC2835671

[B25] Montresor A Crompton DWT Hall A Bundy DAP Savioli L Guidelines for the evaluation of soil-transmitted helminthiasis and schistosomiasis at community level 1998 World Health Organization, Geneva

[B26] WHO-choice Action against worms 2003 1 World Health Organization, Geneva

[B27] WHO Bench aids for the diagnosis of intestinal parasites 1994 World Health Organization, Geneva

[B28] Guzman-Montes GY Ovalles RH Laniado-Laborin R Indirect patient expenses for antituberculosis treatment in Tijuana, Mexico: is treatment really free? J Infect Dev Ctries 2009 3 778 782 20009279 10.3855/jidc.489

[B29] Bratt JH Foreit J Chen PL West C Janowitz B de Vargas T A comparison of four approaches for measuring clinician time use Health Policy Plan 1999 14 374 381 10.1093/heapol/14.4.374 10787653

[B30] Global Productivity Report Global productivity report, a world of unreleased opportunities: proudfoot consulting 2008 http://www.alexanderproudfoot.com/accessed 25 May 2010

